# Pull the Emotional Trigger or the Rational String? A Multi-Group Analysis of Organic Food Consumption

**DOI:** 10.3390/foods11101375

**Published:** 2022-05-10

**Authors:** Qiuqin Zheng, Haimei Zeng, Xintian Xiu, Qiuhua Chen

**Affiliations:** 1 College of Economics and Management, Fujian Agriculture and Forestry University, Fuzhou 350002, China; 2191573001@fafu.edu.cn; 2 Anxi College of Tea Science, Fujian Agriculture and Forestry University, Quzhou 362000, China; 3212333003@fafu.edu.cn; 3 College of Resource and Environment, Fujian Agriculture and Forestry University, Fuzhou 350002, China; xxt200250@fafu.edu.cn

**Keywords:** organic food consumption, positive emotion, sensory appeal, multi-group SEM

## Abstract

The organic food industry in China has been developing fast with the increasing consumer demand for healthier, safer, and more nutritious foods since the epidemic outbreak. It is of great significance to understand the psychological preference of consumers for organic food and adjust the marketing strategy accordingly. In this study, we adopted the multi-group structural equation model (SEM) to analyze 571 questionnaire data and explored the effects of consumers’ perception on the sensory appeal of organic food, perception on promotional stimulation, positive emotion, and perceived social value on the purchase intention of organic food. Based on the Stimulus–Organism–Response (S-O-R) model, this study divides the route affecting organic consumption behavior into the rational route and emotional route. It was proved that the emotional route (positive emotion) has a greater impact on the purchase intention of organic food than the rational route (perceived social value). In addition, there are different purchase intentions among different product types. Specifically, compared with organic tea, positive emotion has a greater effect on the purchase intention for organic rice. This study provides an important reference for the organic food-marketing strategy of enterprises.

## 1. Introduction

In the context of green and sustainable consumption, China and other emerging countries have begun to heavily promote organic consumption in recent years. In 2020, the global sales of organic food and beverage exceeded 120 billion Euros, and China accounted for 10.2 billion Euros (8.5%) [1]. China is the fourth largest organic food market in the world [1]. In 2022, China Central Document No. 1 stated that governments should continue to adjust and optimize the agricultural structure; strengthen the certification and management of green food, organic agricultural products and geographical indications of agricultural products; and increase the supply of high-quality green agricultural products. Compared with traditional food, organic food follows the production standards of organic agriculture, without using chemically synthesized fertilizers, pesticides, growth regulators, and other substances [2,3]. It contains no pesticide residues and does not use growth hormone and genetic engineering (GE) in the growing process, which is more healthy, nutritious, and natural [4]. Organic food plays an important role in promoting environmental protection and agricultural efficiency, enhancing the competitiveness of agricultural products and meeting the demand for safe and high-quality agricultural products. Consumers have linked organic food with health and nutrition since the outbreak of COVID-19. In the next few years, the organic food industry in China has great potential for further expansion [5,6].

Chinese governments have carried out a lot of publicity and education on organic food consumption. With the heavy promotion from governments, consumers are no longer unfamiliar with the concept of “organic.” Yang et al. (2021) [7] found that the subjective cognition level of Chinese consumers on organic food is in a high position. However, the consumption capacity of organic food in China still lags behind other countries [1]. This indicates that only improving consumers’ cognition is not enough. Ignoring the emotional appeal or emotional resonance of individuals cannot effectively promote the real organic consumption behavior of consumers [8]. Food consumption is never just to satisfy the appetite, which is closely related to culture and emotion [9,10,11]. Emotion is the attitude experience of individuals on whether objective things meet their needs. When objective things meet their needs, individuals show a positive attitude and this usually reflects in feelings of love, joy, happiness, etc. [12]. According to the broaden-and-build theory of positive emotions proposed by Fredrickson (1988) [13], various positive emotions such as happiness, interest, satisfaction, pride, and love, are more helpful to expand the scope of attention, cognition, and behavior of individuals; on that basis, people can more effectively obtain and analyze information, make more appropriate action choices, and have the effect of continuously enhancing personal resources for a long time. Moreover, Eastern culture is more emotional compared with the rationality of Western culture [8,14]. Therefore, it is of great significance to explore the role of emotion in organic consumption in China. In addition, the positive emotions of consumers can promote their cognitive flexibility and expand their scope of attention, making consumers aware of the differences between similar products [15,16]. This is also of great significance for brand competition.

Emotion is the most important endogenous factor in individual psychology and plays a very important role in decision-making [11,17]. More and more consumers tend to choose foods that have an emotional resonance with them. Holbrook and Hirschman (1982) [18] were the first to apply emotion to the research field of consumer behavior. They proposed that consumers were not always rational and emphasized the importance of emotion in purchase behavior. For example, Hsu and Tsou (2011) [19] employed the Stimulus–Organism–Response (S-O-R) model, starting from the impact of website quality on the repurchase intention of consumers, to measure consumers’ emotional state through the Mood Scale, and selected it as the intermediary variable. They found that website quality can bring about individual feedback with positive emotions, while the positive emotion is helpful for consumers to repurchase and improve their repeat purchase intention. In marketing practice, especially in advertising design, marketers are increasingly using various emotional experiences to influence consumer behavior and decision-making. Emotion involves the whole process of consumer behavior, i.e., all the behaviors from searching and processing information, to product selection, and then to post-purchase, are closely related to the emotional state of consumers [20,21,22]. Therefore, it is crucial to incorporate emotional factors into the cognitive process of purchase behavior of organic consumers. The analysis of Dispoto (1977) [23] showed that the correlation coefficient between ecological emotion and ecological behavior was 0.15, and many people with little environmental knowledge still show strong emotional loyalty to the environment. This reveals that environmental knowledge and environmental emotion are two independent influencing variables. Kanchanapibul et al. (2014) [24] also considered that it is reasonable to take environmental emotion and environmental knowledge as two different variables to study their impacts on environmental consciousness and behavior.

However, only a few studies are focusing on the impact of emotion on ecological products, low-carbon consumption behavior, and organic products to date. Meneses et al. (2010) [25] analyzed emotional variables and cognitive variables and found that the recycling behavior of consumers was more related to emotional factors than cognitive factors. Based on the study of low-carbon purchase behaviors, Wang and Jing (2012) [26] found that the impact of low-carbon emotion is greater than that of low-carbon knowledge, suggesting that stimulating emotion was more effective than improving cognition in the influence of consumers’ attitude towards low-carbon environmental protection. Lee and Yun (2015) [27] used the S-O-R model to study the two routes of organic consumption, i.e., the emotional route and rational route, and found that consumers are more inclined to cognitive judgment than emotion in purchasing organic food. Jose and Kuriakose (2021) [28] took Indian consumers as the object and compared the effects of emotion, practice, and rationality on organic consumption intention. They found that rational factors such as environmental motivation have little influence, and emotion plays a leading role in organic consumption.

Therefore, to continuously and effectively expand the organic consumption market in China, it is necessary to understand the driving mechanism of the organic food purchase of consumers in China. Based on the S-O-R model and the broaden-and-build theory of positive emotions, this study adopts a multi-group structural equation model (SEM) to explore: (1) Is it the rational route or emotional route that can better enhance the purchase intention of Chinese consumers for organic food? (2) Are there significant differences in the impact routes of different product types (such as organic tea and organic rice)?

## 2. Theoretical Framework and Research Hypothesis

### 2.1. Theoretical Framework

A large number of studies on consumer purchase behavior took the S-O-R model as the theoretical basis since it was proposed by Russell (1974) [29]. The S-O-R model considers that the purchase behavior of consumers is mainly caused by external stimuli (products, situations, etc.), which change the psychological activity of individuals, thereby generating motivation, making purchase decisions, and implementing purchase behavior. Therefore, the S-O-R model can be regarded as a dynamic expression of the purchase behavior process of individuals. Many scholars considered that in this dynamic process, the internal change of the organism is due to the cognition and emotion of individuals to the external stimuli, which will be reflected in the subsequent behavioral response.

**Figure 1:**
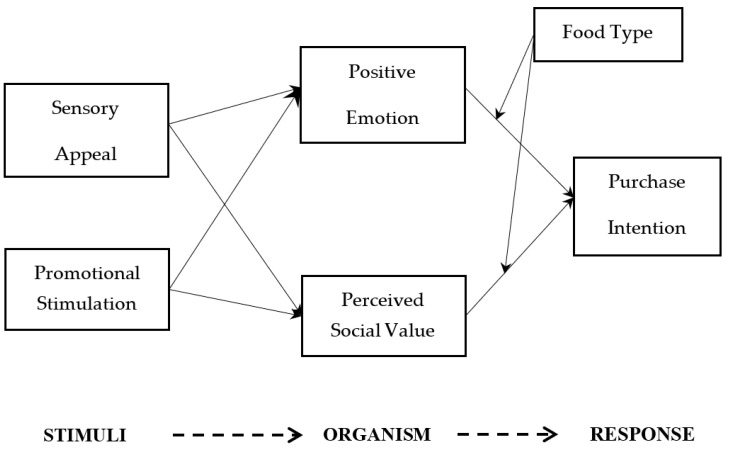
Conceptual model.

One of the main hypotheses of the theory of reasoned action (TRA) and the theory of planned behavior (TPB) is that people are rational in the decision-making process and action; thus, cognitive methods can be used to predict behavior [30]. However, the addition of affective variables has been recommended as a useful extension of the theory [12,28,31]. Following this suggestion and using the S-O-R model, we define the behavioral response as the purchase intention of organic food and divide the internal changes of organisms into two-dimensional dimensions, i.e., emotion and rational (positive emotion and perceived social value), which is helpful to check whether it is the emotional route or the rational route that plays a role in organic consumption behavior in China (Figure 1). Therefore, the S-O-R model is suitable for this study.

Lee and Yun (2015) [27] proposed that it was more suitable to focus on the stimulation of food itself to explore the purchase intention of organic food than psychosocial stimulation, because they believed that the main determinant of the purchase intention of organic food is the product attributes related to health, environmental protection, and animal welfare. However, consumers in China still have little purchasing power for organic food, and they rely more on the publicity of the government and the outside world. Therefore, it is necessary to include both food stimuli (organic food characteristics) and external stimuli (promotional stimulation) in this study.

### 2.2. Research Hypothesis

#### 2.2.1. Sensory Appeal

According to the cue utilization theory [32], consumers usually evaluate products and make purchase decisions based on various internal and external information clues [33,34]. Internal information is inherent in the product itself, including taste and texture, while external information is other information related to the product, such as label, packaging, etc. [35] Acebron and Dopico (2000) [36] pointed out that both internal and external information about products can advance consumers to generate positive emotions and form judgments on product quality, which will further affect their purchase decision of consumers. These can be called sensory attributes [37]. In addition, products with different sensory attributes cause different emotional reactions in consumers [9]. Among these sensory attributes, vision is usually the first sense and overwhelms the perception of other information in attracting consumers’ attention [38]. Visual cues are not only limited to the internal characteristics of the product itself but also involve external characteristics such as product packaging [39]. As the main physical characteristics of food, visual cues not only can indicate the quality of food but also link consumers to other emotional experiences [40,41]. Therefore, they are the attributes with more influence. Lee and Yun (2015) [27] found that consumers’ perception of the sensory appeal of organic food can promote their purchase intention of consumers because organic food brings them positive emotions. Some studies also showed that the sensory attributes of organic food are usually related to pleasure, hedonism, and happiness [42]. Therefore, the following hypotheses are put forward:

**Hypothesis** **1** **(H1):** *Consumers’ perception of the sensory appeal of organic food has a significantly positive impact on the positive emotion of consumers*.

**Hypothesis** **2** **(H2):** *Consumers’ perception of the sensory appeal of organic food has a significantly positive impact on the perceived social value of consumers*.

#### 2.2.2. Promotional Stimulation

Promotional stimulation is a form of communication used to raise consumers’ awareness of the product and can distinguish them from their detractors. It can be used as a source of information to evaluate products and stores [43]. The premise of TRA is that people can completely control their behavioral intention. However, consumers are bound to be interfered with by external factors when making purchase decisions in real life [44]. When the real information about products is scarce or asymmetric, consumers will seek external help to obtain relevant purchase experience or suggestions, and then form the corresponding purchase intention [45]. Marketing promotion can be used as a source of information for evaluating products and stores [43]. The calls of environmental protection associations and governments and advertising factors can stimulate the organic purchase intention of consumers [46]. Chen and Antonelli (2020) [47] considered that external context factors can significantly improve the perceived value and purchase intention of consumers on products. Zhu et al. (2013) [48] found that situational factors of laws and policies can adjust the degree of influence of consumers’ purchase intention on green purchase behavior. Miller et al. (2021) [49] found that situational effects have an impact on the perception and cognitive links of consumers, which can interact with consumers’ psychological perception and then affect their purchase intention. With strong calls from governments, environmental associations, etc., consumers can demonstrate their concern and responsibility for the environment by purchasing and using organic foods, gaining more social recognition and approval [50]. Therefore, the following hypotheses are put forward:

**Hypothesis** **3** **(H3):** *Consumers’ perception of the promotional stimulation of organic food has a significantly positive impact on the positive emotion of consumers*.

**Hypothesis** **4** **(H4):** *Consumers’ perception of the promotional stimulation of organic food has a significantly positive impact on the perceived social value of consumers*.

#### 2.2.3. Positive Emotion

Positive emotion refers to the emotion with a positive valence. It is associated with the satisfaction of certain needs, accompanied by pleasant subjective experience, and can improve the enthusiasm and activity ability of individuals [51]. Gutjar et al. (2015) [10] indicated that the choice of food is mainly related to positive emotions. Meneses (2010) [25] also showed that the recycling behavior of consumers is more based on positive emotion than negative emotion. Based on his research, positive emotions include joy, contentment, interest, pride, gratitude, and love. According to the expansion theory of positive emotion proposed by Fredrickson (2001) [13], positive emotions can advance individuals to break through certain restrictions and produce more thoughts under general conditions, expand the scale of attention, enhance cognitive flexibility, and update and expand the cognitive map of individuals. Isen (2001) [52] found that positive emotions can provide more information for cognitive processing. Plenty of positive experiences with a product may lead to intuitive decisions on future purchases [53]. Existing studies have demonstrated that positive emotions can predict the ecological consumption intention of individuals [54] and also have a significantly positive impact on green purchase behavior and environmental protection behavior [25]. Therefore, the following hypothesis is put forward:

**Hypothesis** **5** **(H5):** *Consumers’ positive emotion has a significantly positive impact on the purchase intention of consumers for organic food*.

#### 2.2.4. Perceived Social Value

Perceived value is the consumer’s preference and evaluation of the product attributes and their utility that helps or hinders the achievement of goals in a given usage context [55]. In the era of consumerism, goods are purchased not only to satisfy individual functional needs but also to achieve the purpose of self-identity construction [56]. Sweeney and Soutar (2001) designed a scale for measuring the perceived value of durable goods, and they viewed perceived social value as the utility of a product to reinforce self-concept. They argued that when customers bought a product, they would consider the impression that the purchase would have on others [55]. Relevant studies from a social perspective have revealed that green consumption behavior stems from personal reputation and status, e.g., people are more willing to pay for environmental protection in public to gain extra points for their image [57,58]. Chinese consumers with collectivist cultural values face more than Western consumers with an individualistic culture [59]. That is, social motives influence Chinese consumers’ green consumption behavior more profoundly than environmental and economic motives. Further, through organic food consumption, consumers can effectively present themselves to others [60]. This is because, compared to convention food, organic food is more expensive and pro-social, which can reflect the non-generic nature of consumers, thus helping them to gain more praise, social recognition, and good impressions [61]. Noppers et al. (2014) [62] stated that consumers brought green products because such consumption helped to project a positive image of themselves. Kohlova and Urban (2020) [63] pointed out that green consumption enhanced consumers’ social status because it helped them to demonstrate wealth-related competencies. People who need to confirm their social status or self-identity will prefer organic food [57]. Therefore, out of rational thinking, consumers will choose organic foods with greater utility in order to demonstrate their social status and value preferences. Therefore, the following hypothesis is put forward:

**Hypothesis** **6** **(H6):** *Consumers’ perceived social value has a significantly positive impact on the purchase intention of consumers for organic food*.

#### 2.2.5. Product Type

Products can be classified as hedonic products and practical products according to different classification standards [64]. Hedonic products can bring emotional joy, while practical products reflect rational cognition [65,66]. In general, practical products are mainly used by consumers to meet some specific tasks and obtain more efficiency, while hedonic products are mainly used to obtain emotional demands, and the consumption is to meet the subjective feeling of consumers [67]. Although these two kinds of products reflect different consumer psychology, they are not opposed to each other. Studies have shown that hedonic attributes and practical attributes have a positive correlation [68]. Some products have both the characteristics of hedonic products and practical products; to be specific, if a product is defined as a hedonic product, it means that the hedonic attributes of this product are greater than the practical attributes, rather than only having the characteristics of hedonic products without any characteristics of practical products [68]. In the situation of different product types, the influence route of purchase intentions of consumers is different [69]. Based on the functional consistency theory, compared with positive emotion, perceived social value emphasizes the practical value of organic food and can help consumers obtain more efficiency, which is matched with the attribute characteristics of practical products and is easy for consumers to generate a positive purchase intention [70]. Based on the self-consistency theory, compared with perceived social value, positive emotion emphasizes the hedonic value, which is matched with the attribute characteristics of hedonic products and is easy for consumers to generate a positive purchase intention [71]. To easily compare the differences in purchase intention between different kinds of organic food, this study defines the tea as the hedonic product and rice as the practical product. Therefore, the following hypotheses are put forward:

**Hypothesis** **7** **(H7):** *In terms of tea, positive emotions have a greater impact on the purchase intention of organic food than perceived social value*.

**Hypothesis** **8** **(H8):** *In terms of rice, perceived social value has a greater impact on the purchase intention of organic food than positive emotion*.

## 3. Methods

### 3.1. Questionnaire Design

The questionnaire is divided into two parts. The first part is the main body of the questionnaire, including the scales of each variable, and the second part is the personal information of the respondents. Assuming that the measurement items of each variable in the model are from the maturity scale that has been widely used in the relevant literature, and have been appropriately modified based on expert opinions and the specific consumption situation of organic agricultural products. The Likert 7-point scale was used as the form of all scales.

As can be see in Table 1, the measurement of sensory appeal (SA) and positive emotion (PE) uses the scale of Lee and Yun (2015) [27]. The measurement of promotional stimulation (PS) uses a modified scale based on the design of Wang et al. (2018) [72], including the publicity of governments and academic institutions, and is suitable for the current mode of promoting organic agricultural products in China. The measurement of perceived social value (PSV) uses the scale of Wang et al. (2017) [8] and Sweeney and Soutar (2001) [55]. The measurement of the purchase intention of organic agricultural products (PI) uses a modified scale based on the design of Kim and Lee (2019) [73].

### 3.2. Data Collection and the Sample

The questionnaire method was used in this study, and the data were collected online based on the professional questionnaire platform (Credamo). In this survey, consumers who purchased organic tea (rice) were taken as the survey object. This is because only those consumers who purchased these products can perceive the relevant organic attributes. Meanwhile, trap questions were set in the questionnaire, i.e., “100 + 100 = ?”. Those who answered wrong were regarded as not seriously filling in the questionnaire.

Before the survey, we conducted a small-scale pilot survey, and a total of 30 pilot survey questionnaires were distributed. Using SPSS24.0, we removed the measurement items with a Cronbach’s α value of less than 0.6. According to the information and suggestions fed back by the pilot survey, the items with unclear semantics and confusion in the questionnaire were adjusted and revised, which can ensure the effectiveness of the questionnaire. In this study, two sets of questionnaires focusing on organic tea and organic rice, respectively, were designed. A total of 571 valid questionnaires were collected, including 290 questionnaires on organic rice and 281 questionnaires on organic tea.

### 3.3. Research Methods

The data analysis in this study was divided into three steps. First, SPSS24.0 and AMOS24.0 were used to test the reliability and validity of variables to ensure the goodness of fit of the structural model. Second, AMOS24.0 was used to conduct a hypothesis test on the structural model to verify the relationship between sensory appeal, promotional stimulation, positive emotion, perceived social value, and purchase intention. Thirdly, the multi-group SEM was used to analyze the regulation of different types of organic foods. The multi-group SEM analysis can explore whether the route model suitable for one sample is also suitable for other samples. Existing studies have mainly focused on single organic food [74], and the studies for comparing and analyzing the differences between the purchase intentions of different types of organic foods are rare.

## 4. Results

### 4.1. Descriptive Statistics Analysis

As shown in Table 2, the number of female samples (55%) is greater than male samples (45%), which is consistent with previous research results in which women are the main buyers of families in China [7,75]. The respondents aged 25~34 account for the largest proportion (62.5%), followed by those aged 35~44, accounting for 22.8%, which means that young consumers show more purchase intention for organic foods. This result is consistent with Chekima et al. (2017) [76] and Yadav and Pathak (2016) [77]. In addition, most respondents had a bachelor’s degree. In terms of family population, families consisting of approximately three to four accounted for the largest proportion, followed by those consisting of approximately five to six people. The monthly income was at the level of 6501 RMB and above. Overall, the survey samples of this study are more in line with the actual situation of organic consumption in China and can be used for further analysis.

### 4.2. Reliability and Validity Test of Samples

The composite reliability (CR) value was used to test the reliability of the questionnaire. From the measurement results of the model in Table 3, the CR values are greater than 0.7, suggesting that the indexes of each dimension have sufficient reliability and internal consistency [78]. The measurement of validity is tested by convergent validity and discriminant validity, in which the convergent validity is mainly reflected by normalized factor loading, Z-value, and average variance extracted (AVE). The results show that the normalized factor loadings are greater than 0.6 and significant, and the AVEs are greater than or close to 0.5, indicating that the scale has high convergence validity [79]. Meanwhile, the correlation coefficient between any two variables is less than the square root of AVE of each variable, as shown in Table 4. Therefore, the scale has good discriminant validity, which lays a foundation for the analysis of the structural model.

### 4.3. Test of the Measurement Model

The measurement model is evaluated using the maximum likelihood method based on AMOS24.0. From Table 5, it can be seen that the overall test results of goodness of fit of the model are χ^2^/df = 2.505, GFI = 0.953, AGFI = 0.932, CFI = 0.966, and RMSEA = 0.051. These indexes of the model meet the standard, indicating that the model fits well.

### 4.4. Test of Structural Equation Model

As shown in Table 6, all hypotheses passed the significance test. As expected, consumers’ perceptions of sensory appeal and promotional stimulation had a significant positive impact on the positive emotion of consumers (β = 0.647, *p* < 0.001 and β = 0.329, *p* < 0.001, respectively). The stronger the consumers’ perception of the sensory appeal of organic food or the stronger the consumers’ perception of external publicity, the higher the positive emotion of consumers. Then, H1 and H2 were supported. Meanwhile, consumers’ perceptions of sensory appeal and promotional stimulation had a significantly positive impact on the perceived social value of consumers (β = 0.505, *p* < 0.001 and β = 0.329, *p* < 0.001, respectively). Then, H3 and H4 were supported. Further, consumers’ positive emotions and perceived social value had a significantly positive impact on the organic purchase intention of consumers (β = 0.579, *p* < 0.001 and β = 0.242, *p* < 0.001, respectively). Then, H5 and H6 were supported.

The Bootstrapping method is used to test the mediating effect. Hayes et al. (2009) suggested that the Bootstrapping method should be repeated at least 5000 times during the mediating effect test. In SPSS 24.0, we adopted the plug-in unit process 4.0 to set the sampling times to 5000 times and the confidence was 95% [80]. The results are shown in Table 7.

The confidence interval (CI) of the indirect effect was used to judge whether the mediating effect exists. If the CI did not include 0, we rejected the original hypothesis, which means that the indirect effect was not 0 and the mediating effect existed [81]. As shown in Table 6, the indirect effect existed and was significant, indicating the existence of the mediating effect; the direct effect was less than the total effect and significant, indicating that there are partial mediating effects.

### 4.5. Multi-Group Analysis

To check whether the route model suitable for the whole sample was also suitable for the specific sample group, and further to check whether different product types have the same influence route [82], this study selected different product types (organic tea and organic rice) as adjustment variables to conduct the multi-group SEM test on the S-O-R model of organic foods. The operation in this part follows the measurement invariance procedure of the composite model proposed by Byrne (2004) [83]. The results are shown in Table 8.

From Table 8, the *p*-value of all competition models is less than 0.05, and the ∆CFI between any two models is less than 0.01, indicating that the multi-group measurement invariance is valid [83]. The research conclusion is applicable to all consumers. Then, the difference in route coefficient between organic tea and organic rice was in-depth investigated, and the results are shown in Table 9.

As can be seen in Table 9, in the four routes of H1–H4, the groups that purchase organic tea and organic rice are both significant at the level of 0.001, and there is no significant difference in route coefficient value and direction. In the route of H5, positive emotion has a significantly positive impact on purchase intention, i.e., β_T_ = 0.382, *p* < 0.001 and β_R_ = 0.714, *p* < 0.001, respectively. In the route of H6, perceived social value has a significantly positive impact on purchase intention, i.e., β_T_ = 0.373, *p* < 0.001 and β_R_ = 0.168, *p* < 0.05, respectively. This shows that the positive emotion of organic rice consumers has a greater impact than that of organic tea consumers on purchase intention; the positive emotion of organic rice consumers has a greater impact than perceived social value. In terms of organic tea, both the positive emotion and perceived social value have a positive impact on purchase intention, and there is no significant difference.

## 5. Discussion

Previous studies have shown that ignoring individual emotions cannot effectively promote the real organic consumption behavior of consumers [12]. The current research mainly focuses on the influencing factors of organic consumption behavior based on the theory of reasoned action (TRA), theory of planned behavior (TPB), motivation-ability-opportunity (MAO) theory, and value-belief-norm (VBN) theory [84]. It is necessary to add emotional variables to enhance the explanatory power of the existing research [12,28,31]. In this study, we adopt the S-O-R model and incorporate rational cognitive factors and emotional cognitive factors for research. By referring to the study of Fredrickson (1998) [13] and Arvola et al. (2008) [85], this study adopts positive emotion rather than negative emotion as the emotional factor. The reason is that food purchase is more based on positive emotions [10]. Our results also confirm this point, that is, in the situation of organic food consumption, positive emotion is a useful influencing factor.

Firstly, compared with the rational route (perceived social value), the emotional route (positive emotion) has a greater effect on the purchase intention of consumers for organic food. Moreover, the positive emotion plays a partial mediating role between the perception of the sensory appeal of organic food and purchase intention, and between the perception of promotional stimulation of organic food and purchase intention. Different from the research of Lee and Yun (2015) [27], this study supports the conclusion of Wang (2015) [12] and Jose (2021) [28]. Specifically, cognition usually features with transience, shallowness, situationally, and low involvement, while emotion is profound and highly involved [12]; if only cognitive education is conducted for consumers without arousing their emotional resonance, it is difficult to turn their cognition into practical behavior. This study believes that external stimuli (sensory appeal and promotional stimulation) can advance consumers to generate a sense of pleasure and satisfaction with their appropriate behavior. They will consciously purchase organic food to maintain and increase this happy emotional experience. The research of Rana and Paul (2017) [86] also showed that the demand of consumers for organic food in developed countries is mainly due to the requirement for meeting their high-level emotional needs, such as respect and self-realization.

Secondly, this study proved that consumers’ perceptions of the sensory appeal of organic food can positively affect the positive emotion and perceived social value of consumers. The increase in the sensory appeal of organic food by one unit increases the positive emotion and perceived social value of consumers by 64.7% and 50.5% respectively. Meanwhile, consumers’ perceptions of promotional stimulation of organic food can positively affect the positive emotion and perceived social value of consumers. The addition to the promotional stimulation of organic food by one unit increases the positive emotion and perceived social value of consumers by 32.9% and 32.9%, respectively. These are consistent with the previous research conclusions [27,28,87], that is, consumers’ choice of organic food is based on complex judgment from perceived external information (such as packaging, price, publicity, etc.) [35]. Based on various internal and external clue information, consumers can form the value judgment or sensory expectation for organic food [36]. In addition, the promotional stimulation of organic food is useful since it can provide consumers with more organic knowledge to help consumers distinguish the positive attributes of organic food from traditional food. Compared with promotional stimulation, consumers’ perception of the sensory appeal of organic food has a greater impact on the positive emotion and perceived social value of consumers [88]. According to the study of Lee and Yun (2015) [27], sensory appeal is usually linked to hedonic attitude and good experience, and the stronger the sensory appeal of organic food, the more pleasant experience it can bring to consumers.

Thirdly, unlike previous studies that focused more on product function and economic value to analyze consumer purchase behavior, this study also reveals the underlying mechanisms that influence organic food consumption from the perspective of perceived social value, further confirming the existence of social motives in organic consumption. In a collectivist culture, where people care more about the connection with people around them, Chinese consumers driven by a sense of face perceive organic food as having higher perceived value. This is consistent with the findings of [14,59]. Therefore, in terms of corporate marketing, the benefits of organic food consumption for others can be promoted so that consumers feel how others around them want them to behave, rather than simply emphasizing the functional or environmental value that organic products bring to consumers.

Finally, based on the multi-group analysis results, there are differences in the relationship between the organic product type and positive emotion and purchase intention. The existing studies are mainly the multi-group analysis of the impact of demographic characteristics or regional differences on consumer behavior [85,89], and studies on the regulation of product types are rare. In this study, we used the multi-group SEM to conduct the test. Although the overall model difference remains unchanged, from the specific impact path, the positive emotion has a greater impact than the perceived social value on purchase intention in terms of organic rice, which is a surprising discovery. This is due to the fact that according to the previous research conclusions, compared with practical products, hedonic products have emotional and symbolic attributes, which match with positive emotions and can form a positive consumer response. Compared with rice, tea has more hedonic properties. One possible explanation for this is that for Chinese consumers, organic tea and organic rice are both hedonic products due to the high price of organic foods [5,6]. Moreover, the price of organic tea is much higher than the price of organic rice. From the perspective of availability, it is easier for consumers to convert their emotions on organic rice to purchase intention. This suggests that organic retailers should reduce the cost of organic food through various channels to reduce the price on one hand and improve the awareness of organic food of consumers to break the price barrier on the other hand.

This study still has the following deficiencies: First, this study only measures the purchase intention of consumers, and there is a big gap between intention and behavior. Future research can perform the measurement of actual purchase behavior. Second, although we choose the positive emotion as the intermediary according to the expansion theory of positive emotion and the characteristics of organic food, there have been some studies to explore the relationship between negative emotion and the purchase intention of consumers [90]. Therefore, negative emotion variables can be added for more systematic comparison in future studies. Third, based on the consumption and price of rice and tea in China, we treat tea as hedonic and rice as utilitarian. This is an empirical judgement that should be confirmed by adding pre-testing in future studies.

## 6. Conclusions

The coronavirus pandemic has intensified consumer demand for healthier, safer, and more nutritious food. Organic food is considered healthier and safer than traditional food, with a self-owned “health halo” [91]. The health needs of consumers will further advance the development of the organic food industry. To successfully satisfy the growing market demand for organic food, marketers and decision-makers should understand the psychological preference of consumers for organic food and adjust marketing strategies accordingly to change their consumption decisions for organic food. This study classifies the routes affecting organic consumption behavior as a rational route and emotional route, and proves the influence of the emotional route (positive emotion) on organic food consumption behavior. Two different kinds of products, i.e., organic tea and organic rice, are taken to conduct the multi-group SEM analysis, and it is found that the product type has a certain impact. In addition, this study also figures out the antecedents of organic food consumption behavior, namely sensory appeal, promotional stimulation, positive emotion, and perceived social value. On that basis, the following suggestions are put forward:

(1) The purchase intention of consumers for organic food is determined by positive emotion, and sensory appeal and promotional stimulation can affect the positive emotion of consumers. Therefore, in the packaging and promotion of organic food, the stimulation and guidance on the positive emotion of consumers should be paid more attention to. Enterprises should provide visual clues for organic food to enhance consumers’ sensory evaluation. The appearance design and product description information design of organic food should also be more concerned.
(2) Try our best to improve the awareness of consumers on organic food. Enterprises can emphasize the positive consequences of sustainable consumption andprovide consumers with systematic information about organic food or organic agriculture. Based on the status-seeking motivation, enterprises can also highlight the prosocial aspect of organic food consumption and show the benefits of organic food to personal social value and environmental protection, thereby continuously improving the organic food consumption.
(3) Perfect the organic consumption market. The organic food consumption market in China still has the problem of generally high prices. The cost of organic food should be reduced through multi-channel strategies. Moreover, organic food manufacturers should strengthen technological innovation, increase production, and reduce production costs, so that consumers can afford healthy and safe organic food.

## Figures and Tables

**Table 1:** The measures.

Variables	Items	Sources
sensory appeal (SA)	Organic tea/rice looks nice	Lee and Yun (2015) [27]
	Packaging of organic tea/rice looks better	
	Organic tea/rice has a pleasant texture	
promotional stimulation (PS)	Government regulation has a big impact on my purchase of organic tea/rice	Wang et al. (2018) [72]
	Government promotion has a great influence on my purchase of organic tea/rice	
	Opinions of experts and academic institutions have a great influence on my purchase of organic tea/rice	
positive emotion (PE)	I will feel happy if I buy organic tea/rice	Lee and Yun (2015) [27]
	I will feel delightful if I buy organic tea/rice	
	I will feel exciting if I buy organic tea/rice	
perceived social value (PSV)	Buying organic tea/rice is good for me	Wang et al. (2017) [8]; Sweeney and Soutar (2001) [55]
	Buying organic tea/rice can form a good impression for me	
	Buy organic tea/rice to get more praise for me	
purchase intention (PI)	I will learn more about organic tea/rice	Kim and Lee (2019) [73]
	I will recommend organic tea/rice to my friends	
	I will continue to choose organic tea/rice in the future	

**Table 2:** Descriptive statistics of consumer social demographic characteristics.

Variables	Definition	Frequency (*n* = 571)	Percentage (*n* = 571)
Gender	Male	257	45%
	Female	314	55%
Marriage	Unmarried	130	22.8%
	Married	441	77.2%
Age	18–24	65	11.4%
	25–34	357	62.5%
	35–44	130	22.8%
	45–54	12	2.1%
	55–64	7	1.2%
	≥65	0	0
Education	Junior high school or below	6	1.1%
	High school (including secondary occupation)	20	3.5%
	College	58	10.2%
	Undergraduate	414	72.5%
	Master or above	73	12.8%
Family member	1–2	28	4.9%
	3–4	362	63.4%
	5–6	165	28.9%
	≥7	16	2.8%
Monthly income (RMB)	≤3500	55	9.6%
	3501–5000	60	10.5%
	5001–6500	72	12.6%
	6501–8000	122	21.4%
	≥8000	262	45.9%

Note: Chinese currency symbols, abbreviated as RenMiBi (RMB).

**Table 3:** Results of measurement model analysis.

Variables	Items	Ustd.	S.E.	Z-Value	P	Std.	SMC	CR	AVE
SA	SA1	1.000				0.728	0.530	0.747	0.497
	SA2	1.004	0.086	11.612	***	0.641	0.411		
	SA3	1.012	0.086	11.707	***	0.741	0.549		
PS	PS1	1.000				0.686	0.471	0.776	0.538
	PS2	1.090	0.084	12.931	***	0.820	0.672		
	PS3	0.898	0.069	13.082	***	0.687	0.472		
PE	PE1	1.000				0.743	0.552	0.81	0.587
	PE2	1.181	0.077	15.367	***	0.810	0.656		
	PE3	1.075	0.071	15.198	***	0.744	0.554		
PSV	PSV1	1.000				0.650	0.423	0.771	0.531
	PSV2	1.200	0.095	12.572	***	0.788	0.621		
	PSV3	1.062	0.084	12.708	***	0.741	0.549		
PI	PI1	1.000				0.824	0.679	0.862	0.676
	PI2	1.036	0.052	19.986	***	0.817	0.667		
	PI3	0.983	0.049	20.106	***	0.826	0.682		

Note: *** *p* < 0.001. Composite reliability (CR), average variance extracted (AVE), sensory appeal (SA), promotional stimulation (PS), promotional emotion (PE), perceived social value (PSV), purchase intention (PI).

**Table 4:** Results of discriminant validity test.

Variables	AVE	PI	PSV	PE	PS	SA
PI	0.676	**0.822**				
PSV	0.531	0.609	**0.729**			
PE	0.587	0.714	0.695	**0.766**		
PS	0.538	0.491	0.542	0.611	**0.733**	
SA	0.497	0.672	0.606	0.750	0.463	**0.705**

Note: The items on the diagonal represent the square roots of the AVE; off-diagonal elements are the correlation estimates. Sensory appeal (SA), promotional stimulation (PS), promotional emotion (PE), perceived social value (PSV), purchase intention (PI).

**Table 5:** Fitting results of model.

Index	Criteria	Model Fit	Result
χ^2^	the smaller the better	207.948	
df	the bigger the better	83	
χ^2^/df	<3	2.505	ideal
GFI	>0.9	0.953	ideal
AGFI	>0.9	0.932	ideal
RMSEA	<0.08	0.051	ideal
CFI	>0.9	0.966	ideal
TLI (NNFI)	>0.9	0.958	ideal

Note: Goodness of fit index (GFI), root mean square error of approximation (RMSEA), standardized root mean square residual (SRMR), comparative fit index (CFI), Tucker–Lewis Index (TLI).

**Table 6:** Results of the hypothesis test.

	Ustd.	S.E.	C.R.	P	Std.	Results
H1: SA → PE	0.67	0.063	10.661	***	0.647	Support
H2: PS → PE	0.286	0.044	6.518	***	0.329	Support
H3: SA → PSV	0.424	0.054	7.828	***	0.505	Support
H4: PS → PSV	0.232	0.041	5.601	***	0.329	Support
H5: PE → PI	0.78	0.085	9.133	***	0.579	Support
H6: PSV → PI	0.402	0.102	3.953	***	0.242	Support

Note: *** *p* < 0.001. Sensory appeal (SA), promotional stimulation (PS), promotional emotion (PE), perceived social value (PSV), purchase intention (PI).

**Table 7:** Results of mediating effect test.

Paths	Total Effect		Direct Effect		Indirect Effect		
	β	T-Value	β	T-Value	β	LLCI	ULCL
SA → PE → PI	0.6296	14.2338	0.323	6.6329	0.3065	0.2171	0.4059
SA → PSV → PI	0.6296	14.2338	0.4497	9.891	0.1799	0.1065	0.264
PS → PE → PI	0.459	10.5131	0.1741	3.9992	0.2849	0.2172	0.3585
PS → PSV → PI	0.459	10.5131	0.2643	5.9661	0.1948	0.1349	0.2594

Note: Sensory appeal (SA), promotional stimulation (PS), promotional emotion (PE), perceived social value (PSV), purchase intention (PI).

**Table 8:** Fit indices for multi-group invariance tests.

Model	χ^2^	DF	P	χ^2^/DF	GFI	AGFI	CFI	RMSEA
Unconstrained	342.943	166	0.000	2.066	0.926	0.893	0.954	0.043
Measurement weights	358.377	176	0.000	2.036	0.923	0.894	0.952	0.043
Structural weights	369.238	182	0.000	2.029	0.920	0.894	0.951	0.043
Structural covariances	371.349	185	0.000	2.007	0.919	0.895	0.951	0.042
Structural residuals	379.028	188	0.000	2.016	0.918	0.895	0.95	0.042
Measurement residuals	431.676	203	0.000	2.126	0.908	0.891	0.94	0.044

Note: Goodness of fit index (GFI), adjusted goodness of fit index (AGFI), comparative fit index (CFI), root mean square error of approximation (RMSEA).

**Table 9:** MGA test results.

Paths	Path Coefficients of Food Type	
	Organic Tea	Organic Rice
H1: SA → PE	0.613 ***	0.678 ***
H2: SA → PSV	0.405 ***	0.535 ***
H3: PS → PE	0.358 ***	0.291 ***
H4: PS → PSV	0.421 ***	0.296 ***
H5: PE → PI	0.382 ***	0.714 ***
H6: PSV → PI	0.373 ***	0.168 *

Note: *** *p* < 0.001; * *p* < 0.1. Sensory appeal (SA), promotional stimulation (PS), promotional emotion (PE), perceived social value (PSV), purchase Intention (PI).

## Data Availability

Not applicable.
